# Normal weight obesity in adolescents: patterns and associated factors

**DOI:** 10.3389/fnut.2025.1637885

**Published:** 2025-07-21

**Authors:** Maria Kaczmarek

**Affiliations:** Institute of Human Biology and Evolution, Faculty of Biology, Adam Mickiewicz University, Poznań, Poland

**Keywords:** normal-weight obesity, adolescents, body composition, eating habits, physical activity, family affluence, place of residence

## Abstract

**Background:**

Normal-weight obesity (NWO), which is defined as excess body fat despite a normal body mass index (BMI), is increasingly recognized as a cardiometabolic risk factor. However, data on its prevalence and associated factors in adolescent populations is limited.

**Objectives:**

To estimate the prevalence of NWO among Polish adolescents and to identify individual and contextual correlates using a multidimensional ecological framework.

**Methods:**

Data were drawn from the cross-sectional, population-based ADOPOLNOR study and included 4,037 adolescents (49.6% boys) aged 10–18 years with a normal BMI, as classified by the International Obesity Task Force (IOTF). Body composition was assessed via bioelectrical impedance analysis. NWO was defined as a fat mass percentage (FM%) at or above the 85th percentile for age and sex, based on national reference values. Logistic regression was used to identify factors associated with NWO.

**Results:**

The prevalence of NWO was 16.5%, which was slightly higher in girls (17.3%) than in boys (15.8%). Adolescents with NWO had significantly higher FM%, fat mass index and fat-to-muscle mass ratio (*p* < 0.001; *r* ≈ 0.57–0.63), highlighting the limitations of BMI in detecting excess adiposity. Multivariate logistic regression analysis revealed that female sex (OR = 1.23, *p* = 0.046), older age (OR = 1.60, *p* = 0.014), family history of obesity (OR = 1.84, *p* = 0.003) and dieting for weight loss (OR = 1.81, *p* = 0.004) were risk factors for NWO. Protective factors included high family affluence (OR = 0.79, *p* = 0.017), high dietary quality (OR = 0.53, *p* < 0.001), regular mealtimes (OR = 0.65, *p* = 0.028) and high physical activity (OR = 0.53, *p* < 0.001).

**Conclusion:**

NWO affects a significant proportion of adolescents and cannot be detected by BMI alone. Routine body composition assessment and the promotion of healthy behaviors are therefore essential for early identification and prevention.

## Introduction

Adolescence, viewed through the lens of life-course health development, is widely regarded as a period of peak health, marked by rapid growth, dynamic physiological and psychosocial change, resilience, and efficient physical functioning (1). However, it is also a time of increased vulnerability to unhealthy risk-taking behaviors that compromise health and quality of life (2). These behaviors include substance use, smoking, violence, unprotected sexual activity, restrictive diets and disordered eating, physical inactivity, inadequate sleep, and exposure to psychosocial stressors such as body image concerns, societal beauty standards, and emotional distress (3–6). Although these behaviors often emerge during adolescence, their effects can persist into adulthood, influencing long-term health trajectories (7). Dahl (8) observed that despite adolescents' physical robustness and resistance to many diseases, mortality rates increase significantly during this period, mainly from behavioral causes. This paradox highlights the complexity of adolescent health, where physical strength coexists with increased exposure to risk (9).

A growing concern in this context is normal weight obesity (NWO), a condition in which individuals have a normal body mass index (BMI) but an elevated body fat percentage (10, 11). Recent studies from different countries have reported that the prevalence of NWO in adolescents with a normal BMI ranges from 8.9% to 55.6%, depending on the population, definitions used and methods of body composition assessment (12). Traditional measures of obesity, such as BMI, do not accurately reflect adiposity or regional fat distribution, particularly visceral fat. As a result, NWO is often overlooked in both clinical and public health practice (13). This ‘hidden' condition is associated with an increased risk of metabolic syndrome, insulin resistance, dyslipidemia and hypertension, all of which are typically associated with overt obesity (14). Consequently, despite not meeting BMI thresholds for overweight or obesity, adolescents with NWO may face similar or even equivalent metabolic risks (15). This highlights both the limitations of BMI as a health indicator and the urgent need for more comprehensive screening tools.

The vulnerability of adolescents to NWO is determined by a complex interplay of biological, psychological and social factors during adolescence period. At the individual level, poor eating habits, low levels of physical activity and sedentary behavior are major contributors to increased body fat (16). Psychological stress, body image concerns and disordered eating behavior, often exacerbated by societal ideals, can further disrupt metabolic health and promote fat accumulation despite a normal BMI. These factors are particularly pronounced in girls, who may engage in weight control behaviors, whereas boys tend to focus on muscle gain and physical performance (17).

In addition to individual behavior, family dynamics play a crucial role in shaping adolescents' health behavior. Parents' attitudes toward diet, physical activity and their own weight status have a significant impact on adolescents' eating patterns and physical activity levels (18). Adolescents who grow up in families where unhealthy habits are normalized are at greater risk of developing NWO. Family health literacy, parenting styles and family structures also influence adolescents' body image perceptions and health behaviors (19).

Socioeconomic and environmental factors further increase the likelihood of developing NWO. Adolescents from lower socioeconomic backgrounds often face barriers to healthy living, including limited access to nutritious food, safe recreational spaces and health care resources (20). Peer relationships may affect lifestyle choices, dietary habits and levels of physical activity, thereby highlighting school environments as a risk factor for NWO (21).

Despite the growing body of research on individual metabolic health markers, the broader context of social and environmental determinants remains underexplored in NWO research. Socioeconomic status, cultural norms and access to healthcare all influence health behaviors and metabolic outcomes, yet these factors are often overlooked in clinical practice. This study aims to address this gap by assessing the prevalence of NWO in adolescents and identifying its key determinants. By examining a wide range of individual, family and environmental factors, the study seeks to provide insights into the multifactorial nature of NWO and to support more effective strategies for its early identification and prevention.

## Materials and methods

### Study design and recruitment

This analysis is based on an observational, cross-sectional survey conducted within the framework of the ADOPOLNOR project, a large-scale research initiative on the health and quality of life of adolescents in Poland. A representative cohort of 10- to 18-year-old students was recruited from primary and secondary schools across Greater Poland using random sampling. The required sample size was estimated using a standard formula for quantitative variables in cross-sectional surveys, following the methodology outlined in a widely accepted guide by Lemeshow and Levy (22). Based on this calculation, a minimum of 550 participants per age group was required, yielding a total target sample size of 4,950 across nine age groups. To account for potential non-response and exclusions, 5,800 adolescents were ultimately selected, representing approximately 1.98% of the 10–18-year-old population in the region.

A stratified, two-stage cluster sampling design was used to ensure a representative sample. In the first stage, 52 public schools were randomly selected from a sampling frame provided by the Ministry of Education via the Poznań Board of Education. Stratification was based on school location (urban or rural), as defined by the Central Statistical Office of Poland. The selected schools encompassed all educational levels and ensured socioeconomic diversity. In the second stage, one to three classes were randomly selected from the target grade within each school, depending on school size and availability. In smaller rural schools, where only one class was available, that class was included. All students from the selected classes were invited to participate. The participants, all ethnically Polish, came from families with diverse social and residential backgrounds representative of the national population. Participation was entirely voluntary.

### Consent, ethics, and data collection

Age-specific consent procedures were implemented in accordance with ethical standards. Adolescents under the age of 16 provided assent alongside written consent from parents or guardians. Those aged 16–17 provided dual consent (both themselves and a parent or guardian), while participants aged 18 or older provided individual written consent. Participation rates were high, with 97.1% of parents and 96.7% of adolescents consenting to participate. Non-participants were not replaced, and no follow-up was conducted; however, the high response rate reduces the likelihood of non-response bias.

The study protocol was approved by the Bioethics Commission of the Medical University of Poznań (Resolution No. 311/07) and was conducted in accordance with the Declaration of Helsinki and its subsequent amendments. Data collection was performed by trained researchers using standardized procedures to ensure data quality and participant safety.

### Anthropometric and body composition measurements

The protocol included medical examinations, anthropometric measurements, and body composition assessments, as well as questionnaires for parents and self-reporting forms. All anthropometric measurements and body composition assessments were performed by well-trained staff following standardized protocols and using regularly calibrated equipment to ensure accuracy and reliability. Standing height and body weight were measured using standard procedures (23) with a portable GPM anthropometer (accuracy 0.1 cm) and a calibrated electronic scale (Radwag 100/200 OW, Radom, Poland; accuracy 0.1 kg). BMI was calculated as weight divided by height squared (kg/m^2^) and used to classify weight status according to Cole and the International Obesity Task Force (IOTF) age- and sex-specific cut-offs (24, 25). Waist circumference (WC) was measured using a non-elastic tape to the nearest 0.1 cm at the midpoint between the rib cage and iliac crest after full expiration (26). Subcutaneous fat was assessed using a standard procedure by measuring skinfold thickness at the triceps, subscapular, and abdominal sites with a calibrated skinfold caliper (accuracy 0.1 cm) and the natural logarithm of their sum (Ln∑SKF) was used for analysis (23).

Whole-body composition was assessed via bioelectrical impedance analysis using a phase-sensitive single-frequency bioelectrical impedance analyser (SF-BIA) (BIA 101, AKERN, Florence, Italy) following standard protocols and the manufacturer's guidelines (https://www.akern.com/en). Measurements were conducted in the morning in a climate-controlled room at an average temperature of 22°C, with participants barefoot and wearing light clothing. Participants were instructed to fast overnight, abstain from eating or drinking for at least 2 h prior testing, avoid physical activity for 12 h beforehand, empty their bladders and remove any metal objects. During the procedure, participants lay supine with their limbs extended and relaxed for at least 10 min. The tetrapolar configuration was used, with low-impedance Ag/AgCl electrodes (Biatrodes, AKERN Srl, Florence, Italy) placed 5 cm apart on the dorsal sides of the hands and feet after the skin had been cleaned with isopropyl alcohol. The analyser operated at 400 μA and 50 kHz and was calibrated biannually using a standard control circuit (Rz = 380 Ω, Xc = 47 Ω), achieving an accuracy of ±1% for Rz and ±2% for Xc. Data were analyzed using BODYGRAM software (version 1.31). For this analysis, specific body composition were selected from the broader range of parameters provided by BIA and included fat mass (FM, in kg and as a percentage), fat-free mass (FFM), body cell mass (BCM), muscle mass (MM) (all in kg), and basal metabolic rate (BMR, in kcal/day). FM, FFM and BCM were standardized by height squared (m^2^) to calculate fat mass index (FMI), fat-free mass index (FFMI), and body cell mass index (BCMI), all in kg/m^2^. The fat mass ratio (FMR) was calculated by dividing the fat mass (FM in kg) by the muscle mass (MM in kg). A higher FMR indicates a greater proportion of body mass as fat compared to muscle, providing an indication of relative adiposity. Central adiposity was estimated using the waist-to-height ratio (WHtR), calculated as waist circumference (in cm) divided by height (in cm).

Normal weight obesity (NWO) was defined as a FM% at or above the 85th percentile for age and sex, despite a BMI within the normal range (27), using internally derived, sex-specific cut-off values from the normal-weight subgroup in the ADOPOLNOR dataset (28). Reference values are presented in Supplementary Table S1.

### Factors

The analysis included a range of individual, family, social and socioeconomic factors. Individual factors included age, sex, eating patterns and physical activity. Age was calculated from birth and examination dates, expressed in decimals and grouped by whole years (e.g. 10.00–10.99 = 10-year-olds). Eating habits were assessed using a Short Food Frequency Questionnaire (FFQ), which demonstrated good internal consistency (Cronbach's α = 0.81). The 15-item questionnaire covered the following categories: staple foods (bread, rice and pasta); fruits and vegetables (fresh fruit and raw or cooked vegetables); protein sources (meat, fish, eggs and dairy); snacks and beverages (cakes, chocolate/sweets and sugary drinks); and eating patterns (breakfast, dinner and supper, as well as dieting for weight loss). Participants reported how often they had consumed each item over the past week or month, depending on the item, using a five-point Likert scale coded as follows: never (=0), 1–3 times per month (=1), 1–2 times per week (=2), 3–4 times per week (=3), or daily (=4). Participants were also asked whether they had followed a weight-loss diet in the past year (No = 0, Yes = 1). Based on these responses, two indices were developed: the Dietary Quality Index (DQI) and the Meal Regularity Index (MRI). The DQI assessed the balance between healthy and unhealthy dietary components, being calculated as the sum of the frequencies of fruit, vegetables, fish/seafood and dairy products, divided by the sum of the frequencies of sweets/desserts and sugary beverages.

A higher DQI score indicates a relatively healthier diet, with greater consumption of nutrient-rich foods and less intake of sugar-dense products.

The formula of DQI was as follows:


$$\begin{array}{r} \text{DQI}=\\ \frac{\left(\text{Frequency of Fruit + Vegetables + Fish},\text{ Seafood + Dairy Products}\right)}{\left(\text{Frequency of Sweets},\text{ Desserts + Sugary Beverages}\right)} \end{array}$$

The MRI reflected the regularity of structured meals relative to snacking behavior, and was calculated by dividing the frequency of breakfast, dinner and supper by the frequency of sweets and sugary beverages according to the following formula:


$$\text{MRI =}\frac{\left(\text{Frequency of Breakfast + Dinner + Supper}\right)}{\left(\text{Frequency of Sweets},\text{ Desserts + Sugary Beverages}\right)}$$

A higher MRI score indicated a more structured eating pattern. Both indices were categorized into tertiles for interpretation. For the DQI, the tertiles represented low (a lower proportion of healthy to unhealthy food intake), moderate (a balanced but suboptimal intake), and high (a greater intake of healthy foods relative to unhealthy ones) dietary quality. For the MRI, tertiles represented irregular meals (snack-dominated), moderately regular meals (partially structured), and highly regular meals (consistent structured meals).

Physical activity (PA) was assessed using the Modifiable Activity Questionnaire for Adolescents (MAQ-A), which records the type, duration and frequency of physical activities performed habitually over the past seven days, including both leisure-time and school-related activities, as well as sedentary behaviors such as watching television and using a computer (29).

Participants reported the frequency and duration of physical activities lasting at least 30 min per day over the past week, including both weekdays and weekends. The total weekly duration of physical activity (in hours) was recorded. Metabolic Equivalent of Task (MET) score was calculated to reflect activity intensity. One MET expresses the energy cost of physical activities relative to resting metabolic rate (RMR); one MET is equivalent to the energy expended while sitting quietly (approximately 3.5 ml/kg/min of oxygen consumption). Each reported activity was assigned a MET value based on the Compendium of Physical Activities, ranging from 0.9 (sleeping) to 18 (running at 10.9 mph) (30). The MET value for each activity was multiplied by the number of hours spent on that activity per day and the number of days it was performed per week, using the formula:


$$\begin{array}{l} \text{MET-hours/week = MET value }\times \text{ duration }(\text{hours/day})\;\\ \;\times \text{ frequency }(\text{days/week}) \end{array}$$

The total weekly MET-hours were then summed across all reported activities to provide an overall estimate of weekly physical activity energy expenditure. Participants were categorized according to activity intensity levels as follows: sedentary behavior (≤1.5 METs), light physical activity (1.6–2.9 METs), moderate physical activity (3.0–5.9 METs), and vigorous physical activity (≥6.0 METs). Based on total weekly MET-hours, participants were further classified into four physical activity levels: inactive (<10 MET-hours/week), low (10–19.9 MET-hours/week), moderate (20–39.9 MET-hours/week) and high (≥40 MET-hours/week). These thresholds align with the World Health Organization's recommendations that adolescents should engage in at least 60 min of moderate-to-vigorous-intensity, mostly aerobic physical activity across the week to get health benefits (31).

Family-level factors, reported by participants' parents via the ADOPOLNOR-R questionnaire, included family history of obesity and hypertension, as well as parental educational attainment. A family history of obesity was defined as the presence of obesity (BMI ≥ 30 kg/m^2^) in at least one parent, based on parental self-report. Participants were classified into two groups: positive family history of obesity (if at least one parent had obesity) and negative family history (if neither parent had obesity). A similar approach was applied to determine family history of hypertension, based on parental self-reported medical records. Parental education, also reported via the ADOPOLNOR-R questionnaire, was categorized as primary/vocational (<12 years), secondary (12 years), or tertiary (>12 years). Socioeconomic status was assessed using the Family Affluence Scale II (FAS II), a student-reported measure that includes four items (number of cars, bedrooms, holidays, and computers in the household). The FAS II yields a score ranging from 0 to 9, which was categorized into three levels of family affluence: low, medium, and high (32).

Social factors included the type of school attended—primary, lower secondary, or upper secondary (general, vocational, or technical) and place of residence. Residence was classified according to DEGURBA framework and population size as follows: (i) rural (<1,000 inhabitants, agriculture-based), (ii) small town (≤25,000), (iii) medium town (25,001–99,999), (iv) large city (≥100,000) (33).

### Data analysis

From the 5,482 ADOPOLNOR participants, only those with a healthy, normal BMI were included in the analysis, resulting in a final sample of 4,037 individuals aged 10 to 18.99 years, comprising 2,005 males (68.8% of the male sample) and 2,032 females (71.9% of the female sample). Descriptive statistics were used to summarize participant characteristics. Sex differences were assessed using Student's *t*-test for normally distributed variables (height, weight, BMI), and the Kruskal–Wallis or Mann–Whitney *U* test for non-normally distributed variables (body composition parameters). Statistical significance was set at α < 0.05. The rank-biserial correlation coefficient (*r*), a standardized effect size appropriate for non-parametric tests, was used to estimate the magnitude of sex differences. Bonferroni correction was applied for multiple comparisons. To identify individual family, and environmental predictors of normal-weight obesity (NWO), logistic regression analysis with backward elimination was applied. To assess multicollinearity, Spearman's rank correlation coefficients (rho) were calculated between all independent variables. All values were below 0.20, indicating no concern. Univariate logistic regression analyses were then performed, and only predictors significantly associated with NWO (*p* < 0.05) were retained for multivariate modeling. Backward elimination was applied to iteratively remove non-significant variables until all retained predictors had *p* < 0.05. The process concluded when the change in parameter estimates between iterations was < 0.001. Model fit was evaluated using the Hosmer–Lemeshow test, where a non-significant result indicates a good model fit. Final results are reported as adjusted odds ratios (ORs) with 95% confidence intervals (CIs). All analyses were performed using IBM SPSS Statistics, Version 29.0 (IBM Corp., Armonk, NY, USA).

## Results

### Participant characteristics

Table 1 presents the basic characteristics of the study participants, both for the total sample and stratified by sex.

**Table 1:** Characteristics of normal weight study participants in the total sample and stratified by sex.

**Characteristic**	**Total sample** ***n*** = **4037**		**Males** ***n*** = **2005**		**Females** ***n*** = **2032**		**M-F Δ *p*^a^**
	***n***	**%**	***n***	**%**	***n***	**%**	
**Sex**							
Male	2,005	49.6					
Female	2,032	50.4					
Age (years), Mean ± SD	14.71 ± 2.54		14.55 ± 2.52		14.84 ± 2.55		0.080
Younger adolescents 10.0 – 12.99	1,203	29.2	613	30.6	591	29.1	
Middle adolescents 13.0 – 15.99	1,390	33.2	695	34.6	695	34.2	
Older adolescents 16.0 – 18.99	1,444	37.6	698	34.8	746	36.7	
**Type of school**							
Primary school	1,217	30.1	615	30.7	602	29.6	0.372
Lower secondary school	1,238	30.7	580	28.9	658	32.4	
Upper secondary school	1,582	39.2	810	40.4	772	38.0	
General (Lyceum)	544	34.4	250	30.8	294	38.1	0.005
Technical school	764	48.3	407	50.3	356	46.1	
Vocational school	274	17.3	153	18.9	122	15.8	
**Place of residence** ^b^							
Village	1,582	39.2	828	41.3	774	38.1	0.056
Small town	809	20.0	407	20.3	396	19.5	
Medium-sized town	853	21.1	415	20.7	433	21.3	
Large city	793	19.7	355	17.7	429	21.1	
**Father's education level**							
Primary/vocational < 12 years	2,058	51.0	1,024	51.1	1,034	50.9	0.195
Secondary 12 years	1,228	30.4	586	29.2	642	31.6	
Tertiary >12 years	751	18.6	395	19.7	356	17.5	
**Mother's education level**							
Primary/vocational < 12 years	1,599	39.7	760	37.9	839	41.3	0.304
Secondary 12 years	1,516	37.5	770	38.4	746	36.7	
Tertiary >12 years	922	22.8	475	23.7	447	22.0	
**Family affluence, FAS II**							
Low	534	13.2	243	12.1	291	14.3	0.163
Medium	1,455	36.2	738	36.8	717	35.3	
High	2,048	50.6	1,024	51.1	1,024	50.4	
Family history of obesity (FHO)	879	21.8	393	19.6	486	23.9	0.176
Family history of HTN (FHHTN)	840	20.8	411	20.5	429	21.1	0.293
Normal weight obesity (NOW)	665	16.5	317	15.8	351	17.3	0.304
**Dietary quality index (DQI)**							
Low quality	436	10.8	247	12.3	189	9.3	0.069
Moderate quality	2,351	58.2	1,166	58.1	1,185	58.3	
High quality	1,250	31.0	592	29.5	658	32.4	
**Meal frequency index (MFI)**							
Irregular meals	465	11.5	162	8.1	303	14.9	**< 0.001**
Moderately regular meals	1,587	39.3	654	32.6	933	45.9	
Highly regular meals	1,985	49.2	1,189	59.3	796	39.2	
**Dieting for weight loss**	835	20.7	142	7.1	693	34.1	**< 0.001**
**Physical activity level (MET-h/week)** ^c^							
Inactive	218	5.5	64	3.2	154	7.6	<**0.001**
Low	1,045	25.9	407	20.3	638	31.4	
Moderate	2,178	53.9	1,145	57.1	1,033	50.8	
High	596	14.7	389	19.4	207	10.2	

^a^*p*-values represent the results of independent samples of Student's *t*-tests for continuous variables and of Pearson's chi-squared tests for categorical variables; bold values indicate statistical significance after Bonferroni correction. ^b^Rural: villages or small communities with a population of fewer than 1,000, where agriculture is the main source of income. Small town: urban areas with a population of up to 25,000. Medium-sized town: urban areas with a population of more than 25,000, but fewer than 100,000. Large city: urban areas with a population of 100,000 or more. ^c^Physical activity (PA) levels are based on weekly energy expenditure in metabolic equivalent task (MET) hours: Inactive (below 10 MET-hours/week), low (10.0–19.9 MET-hours /week), moderate (20.0–39.9 MET-hours /week) and high (40.0+ MET-hours/week).

The sample comprised 4,037 normal-weight adolescents (49.6% male, 50.4% female), with a mean age of 14.71 ± 2.54 years and no significant sex difference (*p* = 0.080). Participants were evenly distributed across the three adolescent age groups: 29.2% aged 10–12.99 (younger), 33.2% aged 13–15.99 (middle), and 37.6% aged 16–18.99 years (older). Residence and school type showed no sex differences, except in upper secondary education, where boys more often attended vocational schools and girls general secondary schools (*p* = 0.005). Parental education and family affluence were similar across sexes with half of parents having primary or vocational education, and about half of the families reporting high affluence. Family histories of obesity (21.8%) and hypertension (20.8%) also did not differ by sex.

### Prevalence and characteristics of normal-weight obesity

**Figure 1:**
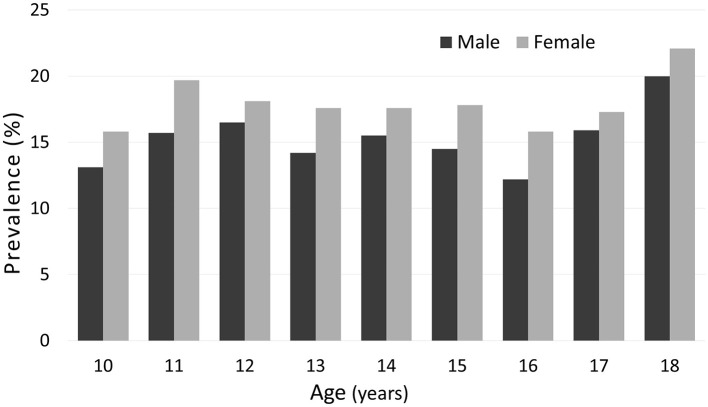
Prevalence of normal-weight obesity (NWO) in adolescents aged 10 to 18 years, stratified by sex and one-year age groups. Sex differences in NWO prevalence at each age were assessed using the chi-squared test. No statistically significant differences were found. Sample sizes by age and sex are provided in Supplementary Table S1.

Normal-weight obesity (NWO) was identified in 16.5% of adolescents, with slightly higher prevalence in girls (17.3%) than boys (15.8%), although the difference was not statistically significant (*p* = 0.304). This sex- and age-related distribution is illustrated in Figure 1.

There are slight fluctuations in the prevalence across age groups for both boys and girls, with a tendency toward a higher prevalence in older adolescents, particularly at age 18, although this tendency is not perfectly steady. No statistically significant sex differences were observed at any age (chi-squared test). Sample sizes by age and sex are provided in Supplementary Table S1.

Girls were more likely to report irregular meals (14.9% vs. 8.1%), dieting (34.1% vs. 7.1%), and low physical activity (31.4% vs. 20.3%), whereas boys were more likely to report regular meals (59.3% vs. 39.2%) and high level of physical activity (19.4% vs. 10.2%) (all *p* < 0.001). Meal frequency, dieting and PA remained significant after Bonferroni correction.

Table 2 shows the median values and interquartile ranges for body composition and anthropometric measurements among NW and NWO adolescents, stratified by sex.

**Table 2:** Median body composition and anthropometric measurements in normal weight and normal-weight obese participants stratified by sex.

**Variable**	**Normal weight**	**Normal-weight obesity**	***p*-value^a^**	**ES *r***
	**Median (Q**_1_ **; Q**_3_ **)**	**Median (Q**_1_ **; Q**_3_ **)**		
Males	*n* = 1689	*n* = 316		
BMI (kg/m^2^)	19.30 (17.62; 20.79)	20.80 (19.38; 22.20)	**< 0.001**	−0.182
FM%	15.40 (11.40; 18.90)	26.40 (24.30; 28.40)	**< 0.001**	−0.621
FMI (kg/m^2^)	2.92 (2.16; 3.70)	5.40 (4.92; 5.95)	**< 0.001**	−0.614
FFMI (kg/m^2^)	16.29 (14.86; 17.72)	15.23 (13.83; 16.51)	**< 0.001**	0.201
BCMI (kg/m^2^)	8.32 (7.32; 9.44)	7.52 (6.66; 8.58)	**< 0.001**	0.188
BMR (kcal/day)	1,648.6 (1,422.2; 1,807.8)	1,684.0 (1,489.4; 1,896.2)	**0.005**	−0.082
FMR	0.28 (0.20; 0.37)	0.58 (0.51; 0.66)	**< 0.001**	−0.605
WHtR	0.41 (0.39; 0.43)	0.43 (0.41; 0.45)	**< 0.001**	−0.248
Ln∑SKF	3.28 (3.06; 3.56)	3.58 (3.23; 3.82)	**< 0.001**	−0.257
Females	*n* = 1,681	*n* = 351		
BMI (kg/m^2^)	19.53 (18.17; 20.95)	21.40 (19.95; 22.70)	**< 0.001**	−0.293
FM%	23.00 (19.00; 26.20)	32.35 (30.60; 33.80)	**< 0.001**	−0.627
FMI (kg/m^2^)	4.40 (3.47; 5.33)	6.94 (6.13; 7.56)	**< 0.001**	−0.568
FFMI (kg/m^2^)	15.10 (14.22; 16.07)	14.38 (13.52; 15.13)	**< 0.001**	0.216
BCMI (kg/m^2^)	7.68 (6.87; 8.45)	7.05 (6.35; 7.64)	**< 0.001**	0.214
BMR (kcal/day)	1,386.2 (1,301.5; 1,452.2)	1,441.9 (1,351.7; 1,497.1)	**< 0.001**	−0.168
FMR	0.48 (0.37; 0.58)	0.77 (0.71; 0.86)	**< 0.001**	−0.629
WHtR	0.41 (0.39; 0.42)	0.43 (0.42; 0.46)	**< 0.001**	−0.308
Ln∑SKF	3.73 (3.45; 3.90)	3.91 (3.70; 4.05)	**< 0.001**	−0.256

^a^*p*-values represent the results of *U* Mann-Whitney test; bold values indicate statistical significance after Bonferroni correction; effect size represents the rank-biserial correlation coefficient *r*.

FM%, fat mass percentage; FMI, fat mass index; FFMI, fat-free mass index; BCMI, body cell mass index; BMR, basal metabolic rate; FMSR, fat-to-muscle mass ratio; WHtR, waist-to-height ratio; Ln∑SKF, natural logarithm of the sum of three measured skinfolds—triceps, subscapula, and abdomen; ES, effect size.

Compared to NW peers, NWO adolescents had significantly higher values for BMI, FM%, FMI, FMR, WHtR, BMR, and Ln∑SKF, and lower values for FFMI and BCMI (all *p* < 0.001, except BMR in boys). Effect sizes (ES *r*) showed large differences in adiposity measures: FM%, FMI, and FMR (*r* ≈ 0.57–0.63), moderate for WHtR and Ln∑SKF (*r* ≈ 0.25–0.31), and small for BMI, FFMI, BCMI, and BMR (*r* < 0.22). These patterns were consistent across sexes, emphasizing that direct fat mass indicators are more effective than BMI or WHtR at distinguishing NWO from NW adolescents.

Significant sex differences (Table 3) were found in most variables within the NWO group (*p* < 0.05), with large effect sizes for FM% (−0.748), FMI (−0.628), and FMR (−0.684); moderate effects for Ln∑SKF (−0.454) and FFMI (0.269) and small effects for BMI (−0.126), BCMI (0.212), and BMR (0.097). WHtR did not differ significantly by sex (*p* = 0.081). Following Bonferroni correction (*p* ≤ 0.0055), all NWO vs. NW differences remained significant. However, BMI and BMR lost significance in sex comparisons within NWO group. This supports the robustness of fat mass indicators.

**Table 3:** Sex differences in body composition and anthropometric measurements in normal-weight obese participants.

**Variable**	**Males *n* = 316**	**Females *n* = 351**	***p*-value^a^**	**ES *r***
	**Median (Q**_1_ **; Q**_3_ **)**	**Median (Q**_1_ **; Q**_3_ **)**		
BMI (kg/m^2^)	20.80 (19.38; 22.20)	21.40 (19.95; 22.70)	0.009	−0.126
FM%	26.40 (24.30; 28.40)	32.35 (30.60; 33.80)	**< 0.001**	−0.748
FMI (kg/m^2^)	5.40 (4.92; 5.95)	6.94 (6.13; 7.56)	**< 0.001**	−0.628
FFMI (kg/m^2^)	15.23 (13.83; 16.51)	14.38 (13.52; 15.13)	**< 0.001**	0.269
BCMI (kg/m^2^)	7.52 (6.66; 8.58)	7.05 (6.35; 7.64)	**< 0.001**	0.212
BMR (kcal/day)	1,684.0 (1,489.4; 1,896.2)	1,441.9 (1,351.7; 1,497.1)	0.045	0.097
FMR	0.58 (0.51; 0.66)	0.77 (0.71; 0.86)	**< 0.001**	−0.684
WHtR	0.43 (0.41; 0.45)	0.43 (0.42; 0.46)	0.081	NA
Ln∑SKF	3.58 (3.23; 3.82)	3.91 (3.70; 4.05)	**< 0.001**	−0.454

^a^*p*-values represent the results of *U* Mann-Whitney test; bold values indicate statistical significance after Bonferroni correction; effect size represents the rank-biserial correlation coefficient *r*.

FM, fat mass percentage; FMI, fat mass index; FFMI, fat-free mass index; BCMI, body cell mass index; BMR, basal metabolic rate; FMS, fat-to-muscle mass ratio; WHtR, waist-to-height ratio; Ln∑SKF, natural logarithm of the sum of three measured skinfolds (triceps, subscapula, and abdomen); ES, effect size.

### Factors associated with normal-weight obesity

Univariate logistic regression analysis (Table 4) identified several factors associated with the likelihood of NWO.

**Table 4:** Univariate logistic regression of associations between normal-weight obesity and demographic, individual and contextual factors in adolescent participants.

**Factor**	**Normal weight obesity**					
	β	**SE**	**Wald** χ^2^	***p*** **-value**	**OR**	**95% CI**
Sex: male (ref.)	0.410	0.216	3.61	0.047	1.37	1.19; 2.30
Age: younger 10–12.99 (ref.)	0.112	0.041	7.29	0.007	1.12	1.03; 1.21
Type of school: primary (ref.)	0.251	0.128	3.85	0.047	1.28	1.07; 1.65
Place of residence: village (ref.)	−0.132	0.096	1.87	0.171	0.88	0.72; 1.06
Father's education: < 12 years (ref.)	−0.270	0.171	2.49	0.115	0.76	0.55; 1.07
Mother's education: < 12 years (ref.)	−0.205	0.097	4.53	0.033	0.81	0.67; 0.98
Family affluence FASII: low (ref.)	−0.333	0.134	6.14	0.013	0.72	0.55; 0.93
Family history of obesity: no (ref.)	0.578	0.253	5.20	0.022	1.78	1.08; 2.93
Family history of HTN: no (ref.)	0.516	0.255	4.09	0.043	1.67	1.01; 2.76
Dietary quality index: low (ref.)	−0.421	0.126	11.19	0.001	0.66	0.51; 0.84
Meal frequency index: irregular (ref.)	−0.238	0.115	4.27	0.039	0.79	0.63; 0.99
Dieting for weight loss: no (ref.)	0.677	0.170	15.93	0.001	1.97	1.41; 2.74
Physical activity: inactive (ref.)	−0.205	0.180	13.19	< 0.001	0.61	0.50; 0.81

β, the log odds coefficient; SE, standard error of the coefficient; Wald χ^2^, Wald chi-square test statistic; *p, p*-value for the Wald test; OR, odds ratio; 95% CI, confidence interval for the odds ratio.

The odds were somewhat higher for girls than boys (OR = 1.37, 95% CI: 1.19–2.30, *p* = 0.047), and increase in age was also associated with an increased risk of NOW (OR = 1.12, 95% CI: 1.03–1.21, *p* = 0.007). School level was borderline significant, with higher school levels associated with increased odds (OR = 1.28, 95% CI: 1.07–1.65, *p* = 0.047). Elevated odds were associated with a family history of obesity (OR = 1.78, 95% CI: 1.08–2.93, *p* = 0.022), hypertension (OR = 1.67, 95% CI: 1.01–2.76, *p* = 0.043), and dieting for weight loss (OR = 1.97, 95% CI: 1.41–2.74, *p* = 0.001). Protective factors included higher maternal education (OR = 0.81, 95% CI: 0.67–0.98, *p* = 0.033), greater family affluence (OR = 0.72, 95% CI: 0.55–0.93, *p* = 0.013), better dietary quality (OR = 0.66, 95% CI: 0.51–0.84, *p* = 0.001), regular meal frequency (OR = 0.79, 95% CI: 0.63–0.99, *p* = 0.039), and physical activity (OR = 0.61, 95% CI: 0.50–0.81, *p* < 0.001). These results highlight the influence of both demographic, familial and lifestyle factors on NWO risk.

Multivariate logistic regression analysis (Table 5) was used to identify independent predictors of NWO, with all covariates adjusted for. The model demonstrated a good fit, as indicated by an adequate level of agreement between the predicted and observed outcomes (Hosmer–Lemeshow test, *p* = 0.087).

**Table 5:** Final multivariate model of factors associated with normal-weight obesity among adolescents: adjusted odds ratios and 95% confidence intervals.

**Factor**	**OR**	**95% CI**	***p-*value for trend**
**Sex**			
Male (ref.)	1		0.046
Female	1.23	1.03; 1.93	
**Age**			
10.0–12.99 (ref.)	1		0.014
13.0–15.99	1.26	1.05; 1.53	
16.0–18.99	1.60	1.10; 2.33	
**Family affluence, FAS II**			
Low (ref.)	1		0.017
Medium	0.82	0.75; 0.90	
High	0.79	0.62; 0.99	
**Family history of obesity**			
No (ref.)	1		0.003
Yes	1.84	1.35; 2.51	
**Dietary quality index**			
Low (ref.)	1		< 0.001
Moderate	0.73	0.62; 0.86	
High	0.53	0.38; 0.74	
**Meal frequency index**			
Irregular (ref.)	1		0.028
Moderately regular	0.81	0.67; 0.98	
Highly regular	0.65	0.45; 0.95	
**Dieting for weight loss**			
No (ref.)	1		0.004
Yes	1.81	1.20; 2.71	
**Physical activity level** ^a^			
Inactive (ref.)	1		< 0.001
Low	0.85	0.61; 0.92	
Moderate	0.65	0.51; 0.79	
High	0.53	0.45; 0.78	

^a^Physical activity (PA) levels are based on weekly energy expenditure in metabolic equivalent task (MET) hours: Inactive (below10 MET-hours/week), low (10.0–19.9 MET-hours/week), moderate (20.0–39.9 MET-hours/week) and high (40.0+ MET-hours/week).

OR, odds ratio; 95% CI, confidence interval for the odds ratio.

Some variables that were significant in the univariate analysis, such as maternal education, family history of hypertension, residence and school type, lost significance in the multivariate model, likely due to confounding or shared variance. Although these factors may indirectly influence adolescent health, they did not independently predict NWO when considering proximal individual- and family-level variables. Female sex remained an independent predictor of NWO (OR = 1.23, 95% CI: 1.03–1.93, *p* for trend = 0.046). Older age groups were also independent predictors of NWO: 13–15 years (OR = 1.26, 95% CI: 1.05–1.53) and 16–18 years (OR = 1.60, 95% CI: 1.10–2.33) vs. 10–12 years (*p* for trend = 0.014). Protective factors included medium (OR = 0.82, 95% CI: 0.75–0.90) and high family affluence (OR = 0.79, 95% CI: 0.62–0.99; *p* for trend = 0.017), better dietary quality (moderate: OR = 0.73; high: OR = 0.53; *p* for trend < 0.001), regular meal frequency (moderately regular: OR = 0.81; highly regular: OR = 0.65; *p* = 0.028), and high PA level (low OR = 0.85; moderate: OR = 0.65; high: OR = 0.53; *p* for trend < 0.001). A family history of obesity remained a strong independent risk factor (OR = 1.84, 95% CI: 1.35–2.51; *p* for trend = 0.003). Notably, adolescents who were dieting to lose weight had almost double the odds of NWO (OR = 1.81, 95% CI: 1.20–2.71; *p* for trend = 0.004), even after adjusting for all other covariates.

## Discussion

This study provides an in-depth analysis of normal weight obesity (NWO) in Polish adolescents, considering biological, behavioral, familial and contextual factors within a population-based framework. By combining precise body composition measurements with a wide range of biopsychosocial variables, the study adds to the growing body of evidence challenging the adequacy of BMI as the sole marker of obesity-related health risks in adolescents. Inspired by Bronfenbrenner's ecological systems theory, we interpret our findings as resulting from the dynamic interaction between individual characteristics, familial surroundings, and wider contextual factors during adolescence, a critical stage of physical and psychosocial development (34).

The prevalence of NWO in this cohort was 16.5% (17.3% in girls and 15.6% in boys), which is consistent with previous regional studies conducted in Kraków, Poland, where rates have ranged from 10% to over 20% over the past decade, varying by sex, age, and year of assessment (35). As outlined in the Introduction, the prevalence of NWO among adolescents varies globally, with different regions experiencing different level of involvement. However, a consistent finding across studies is that BMI alone fails to identify a significant proportion of young people with excess adiposity. García-Hermoso et al. (36) used bioelectrical impedance analysis (BIA) and well-defined cut-off values to report an NWO prevalence of 46% among Colombian eutrophic adolescents aged 9–17 years, applying sex- and age-specific body fat percentage (%BF) thresholds (>23.4–28.3% for boys and >31.0–34.1% for girls). In Japan, Yaguchi-Tanaka et al. (37) found an even higher prevalence of 55.6% among 18-year-old females using a criterion of %BF ≥30%, although the study had a small sample size (*n* = 72). In Brazil, Serrano et al. (38) reported a prevalence of 33.6% among girls aged 14–18 years and 48.7% among those classified as eutrophic by BMI (10–85th percentile) with %BF ≥28%. Based on these findings, Cota et al. (14) assessed 506 Brazilian adolescents with normal BMI (aged 10–19 years) using DXA and reported that 13% met the criteria for NWO. These adolescents were more likely to be female, older, and less physically active, which is consistent with our findings.

In the present study, crude prevalence did not differ significantly between sexes, but interestingly, after adjusting for potential confounders, female sex emerged as a significant predictor of NWO, with girls having 23% higher odds of NWO than boys at equivalent BMI levels. This discrepancy likely reflects confounding and suppression effects, phenomena in which the true relationship between sex and NWO is obscured in unadjusted analyses, but revealed when relevant covariates are controlled for. In this context, adjusting for individual and contextual factors may have revealed a latent association between female sex and an increased risk of NWO. Physiologically, this finding is consistent with well-established pubertal differences in body composition. In girls, puberty is marked by increasing estrogen levels, which promote subcutaneous fat deposition (39). In boys, testosterone enhances muscle mass gain and visceral fat metabolism (40). This physiological divergence likely explains the higher odds of NWO in girls. While behavioral factors may also play a role, the adjusted association is most plausibly attributed to pubertal, hormone-mediated mechanisms affecting body composition (41).

The finding that adolescents with NWO exhibit a metabolically disadvantageous body composition characterized by higher fat mass (FM%, FMI, FMR, WHtR) and lower lean mass (FFMI, BCMI) compared to their NW peers indicates excess adiposity and a lack of metabolically protective lean tissue. This places these individuals at an elevated risk of cardiometabolic disease despite having a normal BMI (14). This pattern aligns with the concept of adiposopathy, which emphasizes that it is not merely the quantity of fat, but its dysfunction, that contributes to metabolic disease (42). Adolescents with NWO may therefore be in a metabolically compromised state, sharing risk profiles with their overweight or obese peers. From a Developmental Origins of Health and Disease (DOHaD) perspective, these early deviations in body composition may lay the groundwork for chronic diseases later in life, particularly when exacerbated by behavioral and psychosocial stressors during adolescence (43).

Among fat-related indices, the fat-to-muscle mass ratio (FMR) shows particular promise for public health screening of adolescents. Zhang et al. (44) demonstrated that the FMR is a useful predictor of dyslipidemia in young adults, supporting its potential for identifying risk factors at an early stage. While most evidence linking FMR to various adverse health outcomes, such as type 2 diabetes, cardiovascular disease, certain cancers and mortality, comes from adult populations (45) these findings highlight the potential long-term significance of elevated FMR in adolescents.

In line with existing literature, older adolescents were found to be at a significantly higher risk of developing NWO (35, 46). This is likely due to the cumulative impact of pubertal hormonal shifts, increasingly sedentary behaviors and the adoption of unhealthy lifestyle patterns, all of which often intensify during late adolescence. Behavioral factors, particularly those affecting energy balance, emerged as pivotal determinants. Protective behaviors included adhering to a high-quality diet, maintaining regular mealtimes and engaging in moderate-to-vigorous physical activity (MVPA). These patterns align with both the Energy Balance Model, which emphasizes the role of behavior in maintaining energy homeostasis, and Self-Determination Theory, which highlights the importance of intrinsic motivation in sustaining engagement in health-promoting activities such as physical exercise (47, 48).

Of particular concern is the sex disparity in these behaviors. Adolescent girls reported lower levels of physical activity and more irregular eating patterns, which may exacerbate their biological predisposition to fat accumulation and make them more vulnerable to NWO. From a circadian and metabolic regulation perspective, irregular mealtimes may disrupt hormonal balance and energy metabolism, thereby further elevating risk. Additionally, a striking and counterintuitive finding was the strong association between dieting for weight loss and an increased risk of NWO. Adolescents who reported dieting were almost twice as likely to be affected, suggesting that restrictive eating, weight cycling and psychological stress, particularly among girls, may paradoxically promote fat gain and muscle loss. This pattern supports the ‘thin outside, fat inside' (TOFI) profile and calls into question the effectiveness of unsupervised weight control strategies (49). From an ecological systems perspective, these behaviors do not emerge in isolation. Rather, they are influenced by family norms, school environments, peer dynamics and broader cultural messages about body image and health. These multi-level influences, operating across Bronfenbrenner's micro- and meso-system levels, emphasize the need to consider the living environment when designing preventive interventions for adolescent health behaviors.

At the family level, a history of obesity emerged as a strong predictor of NWO. This finding is consistent with ecological systems theory and epigenetic research indicating the intergenerational transmission of metabolic risk via shared genes and environments conducive to obesity (50, 51). Higher family affluence was protective, in line with literature on social determinants of health, which suggests that economic capital affords access to healthier food options, opportunities for physical activity, and lower exposure to chronic stress (52). Although maternal education showed a protective trend in univariate analysis, it lost significance in multivariate models, suggesting that economic rather than cultural capital may be more influential in this adolescent population (53).

Although residential setting and school type were not significant factors in the final model, they remain theoretically relevant. Urban living and high-track schools are often associated with sedentary lifestyles, screen time and academic stress, which are factors known to influence energy balance and psychosocial well-being (54). These influences correspond to the macrosystem level in ecological models and warrant further exploration using more detailed environmental exposure data, such as neighborhood walkability or school health policies.

## Implications for screening and public health

This study highlights the limitations of using BMI as the sole indicator of adolescent health. Individuals with NWO may have elevated body fat despite having a normal BMI. These findings support the integration of body composition assessments into routine adolescent health evaluations, particularly within primary care and school health programs. Adopting a multidimensional, ecological approach that considers biological, behavioral, familial and socioeconomic factors can help identify at-risk youth earlier and more effectively. Screening for body composition, particularly among girls and adolescents from less affluent backgrounds, could inform targeted health education and prevention strategies, even in the absence of overt metabolic symptoms. Early identification and intervention are crucial, as NWO has been associated with an increased risk of cardiometabolic disease later in life. Public health initiatives should therefore consider incorporating body composition monitoring and bespoke lifestyle interventions, such as nutrition education and physical activity promotion, into adolescent health surveillance and equity-focused strategies. Furthermore, policy initiatives should strive to reduce disparities by enhancing access to preventive care and health-promoting resources in underserved communities.

## Study limitations

This study has several limitations that should be noted. Recall bias may have affected the accuracy of self-reported exposure and outcome data, which is a well-documented issue in similar research and involved in the premises of this study (55). This is particularly relevant in the context of the two instruments used in this study—MAQ-A and FFQ. Both are widely validated and practical for large-scale epidemiological research. The MAQ-A provides estimates of energy expenditure (in MET-hours per week) and is valued for its low cost, ease of administration, and ability to capture structured physical activity patterns in adolescents. Using the 7-day recall format rather than the traditional 12-month version likely reduced long-term recall error and better reflected behavior relevant to the assessed health status. Nevertheless, it may not fully capture habitual activity levels or seasonal variation, and no objective validation using accelerometers was conducted. The FFQ provides a practical method of assessing general dietary patterns in large populations, with less burden on participants than detailed dietary records. Although it does not provide precise nutrient quantification and is subject to misreporting, particularly among adolescents, it remains a standard tool in nutritional epidemiology. Taken together, these instruments enable the collection of meaningful behavioral data within the logistical and financial limitations of large scale, field-based studies.

In terms of body composition, although bioelectrical impedance analysis (BIA) is less accurate than imaging methods such as dual-energy X-ray absorptiometry (DEXA), its strong correlation with DEXA and and portability make it a suitable alternative for large-scale adolescent health surveillance (56). Furthermore, the study did not include a direct assessment of physical changes during puberty (Tanner staging), but given the fact that the general pattern of maturation correlates with chronological age, age can serve as a reasonable proxy (57). Socioeconomic status was not measured using per capita income, however, family wealth is considered a more relevant indicator of household purchasing power (58). The use of internal reference data from the ADOPOLNOR study to define body fat cut-offs improves the cultural and population specificity of the findings, although it reduces direct comparability with international studies. Despite these limitations, the study has notable strengths. It includes a large, representative, non-clinical adolescent sample, thereby improving generalizability within the national context. While the cross-sectional design limits causal inference, it is well-suited to surveillance studies aimed at identifying associations, such as those between NWO and individual, family and contextual factors and informing future longitudinal research (59). The integrative perspective, combining biological, behavioral and environmental data, adds depth to the analysis. Finally, using multiple adiposity indices in combination with BIA improves the reliability and validity of NWO classification.

## Conclusions

This study improves our understanding of adolescent obesity by identifying NWO as a distinct and frequently overlooked metabolic risk factor. NWO poses a hidden threat that can have serious long-term health consequences. Adopting an ecological-developmental approach, the study identifies key individual and contextual factors that contribute to this condition. The findings support the inclusion of body composition screening in routine adolescent health assessments, particularly in school and primary care settings. Prevention efforts should prioritize physical activity, healthy nutrition and positive body image, particularly among girls and adolescents from socioeconomically disadvantaged backgrounds.

Further longitudinal research is required to establish the causal pathways involved and to evaluate the effectiveness of targeted interventions. Further studies should also investigate the biological mechanisms underlying sex differences in fat distribution and NWO development.

## Data Availability

The data generated and/or analyzed during the current study are available from the corresponding author upon reasonable request, due to privacy concerns.
