# Supplementary material for: Normal weight obesity in adolescents: patterns and associated factors
Source: Front Nutr. 2025 Jul 21;12:1637885. doi: 10.3389/fnut.2025.1637885 (PMC12320748; doi:10.3389/fnut.2025.1637885)
Supplement: Supplementary file 1 [file Table_1.docx]

Table S1. Centile reference values for fat mass percentage in normal-weight Polish adolescents aged 10 to18

| Age | Sample | LMS parameters | |  | Fat mass percentage Centiles | | | | | | |
| --- | --- | --- | --- | --- | --- | --- | --- | --- | --- | --- | --- |
| (years) | size, n | L | S | 3rd | 10th | 25th | M 50th | 75th | 85th | 90th | 97th |
|  | 2005 |  | Males | | | | | | | | |
| 10.0 | 216 | 0.571 | 0.323 | 8.97 | 11.79 | 14.97 | 18.89 | 23.19 | 24.62 | 27.38 | 31.81 |
| 10.5 |  | 0.581 | 0.324 | 8.81 | 11.61 | 14.78 | 18.66 | 22.92 | 24.52 | 27.06 | 31.42 |
| 11.0 | 207 | 0.593 | 0.325 | 8.61 | 11.40 | 14.54 | 18.38 | 22.59 | 24.43 | 26.67 | 30.96 |
| 11.5 |  | 0.603 | 0.326 | 8.45 | 11.22 | 14.34 | 18.15 | 22.31 | 24.38 | 26.34 | 30.57 |
| 12.0 | 212 | 0.613 | 0.327 | 8.28 | 11.03 | 14.12 | 17.90 | 22.01 | 24.30 | 25.99 | 30.16 |
| 12.5 |  | 0.623 | 0.328 | 8.10 | 10.83 | 13.90 | 17.63 | 21.69 | 24.11 | 25.61 | 29.72 |
| 13.0 | 226 | 0.633 | 0.329 | 7.92 | 10.63 | 13.67 | 17.36 | 21.36 | 23.72 | 25.22 | 29.26 |
| 13.5 |  | 0.644 | 0.330 | 7.71 | 10.39 | 13.40 | 17.04 | 20.98 | 23.31 | 24.76 | 28.72 |
| 14.0 | 226 | 0.654 | 0.331 | 7.54 | 10.21 | 13.18 | 16.78 | 20.67 | 22.85 | 24.40 | 28.30 |
| 14.5 |  | 0.664 | 0.332 | 7.38 | 10.03 | 12.98 | 16.55 | 20.39 | 22.41 | 24.07 | 27.91 |
| 15.0 | 209 | 0.674 | 0.333 | 7.23 | 9.87 | 12.80 | 16.34 | 20.14 | 22.01 | 23.78 | 27.56 |
| 15.5 |  | 0.686 | 0.335 | 7.08 | 9.71 | 12.63 | 16.13 | 19.90 | 21.63 | 23.49 | 27.22 |
| 16.0 | 237 | 0.696 | 0.336 | 6.97 | 9.60 | 12.51 | 16.01 | 19.75 | 21.32 | 23.32 | 27.01 |
| 16.5 |  | 0.706 | 0.337 | 6.88 | 9.52 | 12.43 | 15.92 | 19.66 | 21.36 | 23.20 | 26.87 |
| 17.0 | 222 | 0.716 | 0.338 | 6.81 | 9.46 | 12.39 | 15.89 | 19.62 | 21.42 | 23.16 | 26.81 |
| 17.5 |  | 0.726 | 0.339 | 6.75 | 9.43 | 12.37 | 15.89 | 19.63 | 21.91 | 23.17 | 26.82 |
| 18.0 | 250 | 0.738 | 0.340 | 6.70 | 9.41 | 12.39 | 15.92 | 19.68 | 22.42 | 23.24 | 26.89 |
| 18.5 |  | 0.748 | 0.341 | 6.67 | 9.41 | 12.41 | 15.97 | 19.75 | 22.45 | 23.31 | 26.97 |
|  |  |  |  | | | | | | | | |
|  | 2032 |  | Females | | | | | | | | |
| 10.0 | 216 | 0.542 | 0.295 | 10.50 | 13.30 | 16.45 | 20.30 | 24.52 | 27.32 | 28.63 | 32.97 |
| 10.5 |  | 0.630 | 0.287 | 10.63 | 13.53 | 16.72 | 20.55 | 24.66 | 27.14 | 28.60 | 32.69 |
| 11.0 | 228 | 0.736 | 0.278 | 10.82 | 13.84 | 17.09 | 20.90 | 24.91 | 26.92 | 28.67 | 32.51 |
| 11.5 |  | 0.825 | 0.270 | 11.01 | 14.15 | 17.46 | 21.26 | 25.20 | 27.21 | 28.83 | 32.50 |
| 12.0 | 203 | 0.913 | 0.263 | 11.24 | 14.50 | 17.87 | 21.68 | 25.55 | 27.52 | 29.08 | 32.60 |
| 12.5 |  | 1.002 | 0.256 | 11.50 | 14.89 | 18.33 | 22.15 | 25.96 | 28.03 | 29.40 | 32.79 |
| 13.0 | 208 | 1.090 | 0.249 | 11.78 | 15.31 | 18.81 | 22.64 | 26.40 | 28.54 | 29.76 | 33.03 |
| 13.5 |  | 1.197 | 0.241 | 12.13 | 15.81 | 19.39 | 23.22 | 26.93 | 28.95 | 30.19 | 33.34 |
| 14.0 | 217 | 1.285 | 0.234 | 12.42 | 16.24 | 19.87 | 23.70 | 27.36 | 29.35 | 30.54 | 33.59 |
| 14.5 |  | 1.373 | 0.228 | 12.73 | 16.66 | 20.34 | 24.16 | 27.77 | 29.61 | 30.87 | 33.83 |
| 15.0 | 250 | 1.462 | 0.222 | 13.04 | 17.08 | 20.80 | 24.60 | 28.16 | 29.92 | 31.19 | 34.06 |
| 15.5 |  | 1.568 | 0.214 | 13.42 | 17.57 | 21.33 | 25.11 | 28.60 | 30.25 | 31.55 | 34.31 |
| 16.0 | 258 | 1.657 | 0.209 | 13.75 | 17.98 | 21.75 | 25.51 | 28.94 | 30.53 | 31.82 | 34.50 |
| 16.5 |  | 1.745 | 0.203 | 14.09 | 18.39 | 22.18 | 25.91 | 29.28 | 30.85 | 32.09 | 34.69 |
| 17.0 | 209 | 1.834 | 0.197 | 14.45 | 18.83 | 22.62 | 26.33 | 29.65 | 31.24 | 32.39 | 34.92 |
| 17.5 |  | 1.922 | 0.192 | 14.86 | 19.29 | 23.10 | 26.78 | 30.05 | 31.74 | 32.73 | 35.20 |
| 18.0 | 243 | 2.028 | 0.186 | 15.38 | 19.88 | 23.71 | 27.36 | 30.57 | 32.34 | 33.20 | 35.60 |
| 18.5 |  | 2.117 | 0.181 | 15.84 | 20.40 | 24.23 | 27.85 | 31.03 | 32.72 | 33.61 | 35.95 |

Data are derived from Kaczmarek et al. (28). Abbreviations: L, lambda - Box-Cox transformation; M, median; S, sigma - coefficient of variation.
